# Characterizing bacterial communities in paper production—troublemakers revealed

**DOI:** 10.1002/mbo3.487

**Published:** 2017-05-14

**Authors:** Anita Zumsteg, Simon K. Urwyler, Joachim Glaubitz

**Affiliations:** ^1^ Omya International AG R&D Microbiology Oftringen Switzerland

**Keywords:** biofilms, diversity, indicators, metagenomics, microbial contamination

## Abstract

Biofilm formation is a major cause of reduced paper quality and increased down time during paper manufacturing. This study uses Illumina next‐generation sequencing to identify the microbial populations causing quality issues due to their presence in biofilms and slimes. The paper defects investigated contained traces of the films and/or slime of mainly two genera, *Tepidimonas* and *Chryseobacterium*. The *Tepidimonas* spp. found contributed on average 68% to the total bacterial population. Both genera have been described previously to be associated with biofilms in paper mills. There was indication that *Tepidimonas* spp. were present as compact biofilm in the head box of one paper machine and was filtered out by the paper web during production. On the other hand *Tepidimonas* spp. were also present to a large extent in the press and white waters of two nonproblematic paper machines. Therefore, the mere presence of a known biofilm producer alone is not sufficient to cause slimes and therefore paper defects and other critical factors are additionally at play. For instance, we identified *Acidovorax* sp., which is an early colonizer of paper machines, exhibiting the ability to form extracellular DNA matrices for attachment and biofilm formation.

## 1 Introduction

Paper manufacturing requires a large volume of water, which, today, is permanently recycled at the various stages during the production process. As such, bacterial growth and biofilm formation in the paper machines are inevitable. These recycled waters are a main cause of slime production related to the presence of bacteria which leads to smell, discoloration, and irregularities in the paper formation and web breaks (Blanco, Negro, Gaspar, & Tijero, 1996; Kolari, 2007). To mitigate these effects the microbial population is continuously treated with biocides (Blanco, Negro, Monte, Fuente, & Tijero, 2004). But when bacterial colonization is out of control, the consequences are variable paper quality, increasing down time, and higher maintenance costs (Kolari, Nuutinen, Rainey, & Salkinoja‐Salonen, 2003).

Various bacterial species may be responsible for biofilm formation in paper machines*. Deinococcus geothermalis* is a primary colonizer leading to thick, synergistic biofilms with different bacilli species (Kolari, Nuutinen, & Salkinoja‐Salonen, 2001). Furthermore, *Tepidimonas* spp., belonging to the Betaproteobacteria, were identified directly in the paper process already at the early stage of biofilm formation (Tiirola, Lahtinen, Vuento, & Oker‐Blom, 2009). Several bacterial classes and genera are known to populate the waters and raw products in paper machines. Vaisanen et al. (1998) analyzed 390 cultivable aerobic bacteria from process steps and raw materials and demonstrated a vast bacterial diversity. A thorough phylogenetic analysis of 404 cloned 16S rRNA gene amplicons was performed by Granhall et al. (2010), who analyzed two different paper mills that showed similar overall profiles but still unique individual populations. Bacteroidetes (including the genus *Chryseobacterium*) predominated, but several other Phyla were identified such as members of the Firmicutes (including *Clostridium* sp.), Alpha—and Gammaproteobacteria, but not Betaproteobacteria.

Most of the published research focuses on cultivable bacteria from smoothly running paper machines. However, in this study we use, to our knowledge for the first time, Illumina next‐generation sequencing to analyze the total bacterial community, including the uncultivable bacteria, to compare the communities present in the process waters of four paper machines at the same mill. The exemplified paper mill in this report experienced recurring problems in one of the four paper machines. We identified and compared the bacterial population found directly in the irregularities on the paper sheets consistently produced by this machine. Such a thorough process analysis allows us to identify process steps harboring the problematic microbial populations, and thus, in principle, enabling a more efficient strategy to be followed in the future for their control.

## 2 Materials and Methods

### 2.1 Sampling and enumeration of cultivable bacteria

**Figure 1:**
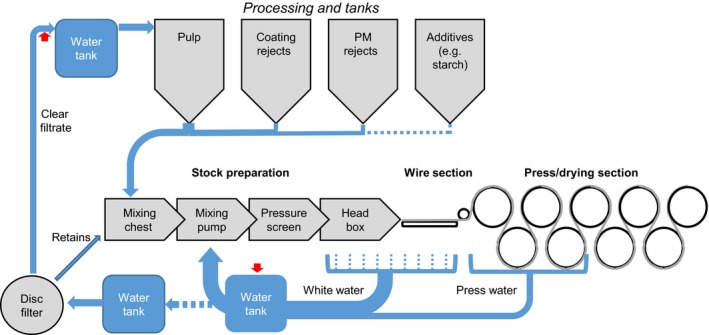
Simplified scheme of water circulation in a typical paper machine displaying the three sampling points: clear filtrate, white water, and press water. Red arrows indicate sites of biocide addition. Remark: waters from the clear filtrate water tanks of all paper machines are used for pulping

All samples were provided from a northern German paper manufacturer (undisclosed) and are listed in Table S1. Defective paper samples were derived from paper machine 1 (PM1). Additionally, waters (press water, white water, and clear filtrate) were sampled from all four paper machines (PM1, PM2, PM3, and PM4) located at the same site. Figure 1 represents a simplified scheme of the process and water circulation, and illustrates the three types of water (press water, white water, and clear filtrate) sampled. The total viable count (TVC) of water samples was determined by plating 0.1 ml of a 10‐fold dilution in phosphate buffered saline (PBS) (pH 7.4, Sigma‐Aldrich) onto Tryptic Soy Broth Agar (TSA) (Sigma‐Aldrich). Plates were incubated for 48 h at 30°C prior to enumeration of colony‐forming units (cfu). Counts with 1–9 cfu/plate and 10–99 cfu/plate were reported as >10^2^ cfu^.^ml^−1^ and >10^3^ cfu^.^ml^−1^, respectively. Higher counts were reported as >10^4^ cfu^.^ml^−1^ when colonies remained separated or >10^5^ cfu^.^ml^−1^ when colonies fused to bacterial lawns. No bacterial viable count was done for paper samples.

### 2.2 Propidium monoazide treatment and DNA extraction

For better accessibility of bacteria in slurries, bacteria were separated from turbid insoluble compounds, such as minerals and pigments, using density gradient centrifugation. For this, 1‐ml water samples were overlaid onto 0.3 ml of 1.6‐mol^.^l Histodenz^™^ (Sigma‐ Aldrich) in 2‐ml microcentrifuge tubes and centrifuged at 10 000 rcf (relative centrifugal force; 1 rcf = 9.81 m^.^s^−2^) for 6 min with slow deceleration. The upper phase, including the interphase was pelleted in a new tube at 10,000 rcf for 3 min. For propidium monoazide (PMA) treatment (Nocker, Cheung, & Camper, 2006), the pellet was resuspended in 0.5‐ml sterile PBS and PMA added to a final concentration of 0.05 mmol^.^l, placed on ice and exposed to a 500 W halogen light source for 4 min to cross‐link the PMA with the free DNA. This ensures that DNA from dead cells is not amplified in the following PCR reaction. The PMA‐treated samples were then pelleted again. From these final pellets, DNA was isolated using the DNeasy Blood & Tissue Kit (QIAGEN, Hilden, Germany) according to the manufacturer's instructions.

To identify the causative bacterial community for the defect paper, we also analyzed the bacterial population present at the defect sites on the paper sheets. For these paper samples, DNA was isolated using the PowerSoil^®^ DNA Isolation Kit (MO BIO Laboratories, Inc., Carlsbad, USA) also according to the manufacturer's instructions.

### 2.3 Bacterial DNA quantification

DNA was quantified by real‐time PCR targeting the 16S rRNA gene as described previously (Clifford et al., 2012). Briefly, in a 25‐μl final reaction volume the primer pair rtPCR_f (ACTCCTACGGGAGGCAGCAGT) and rtPCR_r (TATTACCGCGGCTGCTGGC) were used (Clifford et al., 2012) at 500 nmol^.^l, 10% (v/v) of template DNA, and FastStart SYBR Green Master Mix (Roche cat. No. 4673484001). Using the Thermocycler RotorGene (Qiagen) and the sequential thermal profile (1) 10 min at 95°C followed by (2) 45 cycles of 20 s at 95°C, 56°C, and 72°C, the concentration of bacterial DNA was quantified relative to a DNA standard curve consisting of a known concentration of *Escherichia coli* K1 genomes (approx. 3000 16s rRNA copies per μl).

### 2.4 16S rRNA amplicon sequencing and data analysis

For library generation the V3 and V4 region of 16S rRNA region was amplified by PCR with 30 cycles from the extracted DNA. PCR protocol, primer, and library generation were performed exactly as described by (Illumina (2013) using MiSeq Reagent Kit v3 600‐cycles (Illumina, San Diego CA., Cat. No. S102‐3003). Data were acquired using the MiSeqDx System MiSeq and metagenomic analysis of the raw data was performed using the in‐system software MiSeq Reporter. For taxonomic classification, the Greengenes Database files were used (Mc Donald et al., 2012). In Greengenes an OTU refers to the terminal level at which the sequence is classified.

## 3 Results and Discussion

The exemplified paper mill experienced recurring problems in one of the four paper machines (PM1). The final paper showed defects in terms of irregular spots and holes of approximately 1 cm diameter due to slime deposits in the web during continuous line production. Consequently, the machine had to be stopped and cleaned more frequently than the other paper machines (PM2, PM3, and PM4) leading to costly down time and maintenance. Biofilms have been described as a reason for such slimes and consequently the resulting paper defects (Lahtinen, Kosonen, Tiirola, Vuento, & Oker‐Blom, 2006).

**Figure 2:**
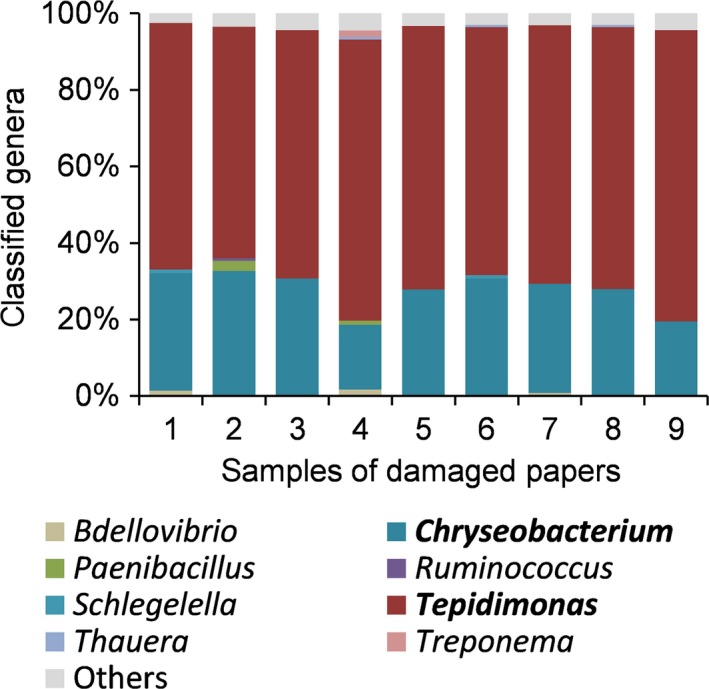
Bacterial population, identified by 16S rRNA metagenomics analysis, at sites of damage in the final paper of PM1

To identify the causative bacterial community we analyzed the bacterial population present at the paper defect site. The DNA was isolated from the paper samples and the amount of bacterial DNA quantified by 16S rDNA PCR (Table 1). All paper samples contained a high amount of bacterial DNA equivalent to approximately 10^5^ to 10^6^ *Escherichia coli* genomes per cm^2^. Using the purified DNA, the bacterial population was further characterized and quantified by Illumina 16S rRNA metagenomics analysis (Illumina, 2013). Interestingly, all nine samples analyzed showed the exact same genus distribution with two extraordinarily predominant genera; *Tepidimonas* and *Chryseobacterium* (Figure 2). These two genera represented at least 90% (average 95%) of all classified genera in all paper samples analyzed, whereby *Tepidimonas* contributes by far the majority with at least 60% (average 68%). Out of the more than 80 *Chryseobacterium* species that exist (Parte, 2014), only one *Chryseobacterium soli* was found here. For *Tepidimonas* four different species out of five known to date were found (Albuquerque, Tiago, Veríssimo, & Da Costa, 2006). *Tepidimonas* has been associated previously with biofilms in different paper mills (Tiirola et al., 2009). Particularly at early stages of biofilm formation, this genus represented more than 40% of the population as quantified by length‐heterogeneity PCR analysis of 16S rRNA (Tiirola et al., 2009). The other genus, *Chryseobacterium*, and related genera from the Bacteroidetes have been identified by T‐RLFP in biofilms of paper mills (Granhall et al., 2010) and they have been described to form slimes (Oppong, King, & Bowen, 2003). Our data point toward *Tepidimonas* spp. and *Chryseobacterium* sp. as causative agents for the defects in the paper sheets. It was very surprising, though, that the bacterial diversity in the samples was extremely low, reduced to mainly these two genera.

**Table 1:** Quantification of bacterial contents in paper samples by 16S real‐time PCR relative to a standard consisting of genomic DNA equivalents of *E. coli* K1

Paper sample no.	Genome equivalents cm^−2^
1	2·10^6^
2	5·10^5^
3	5·10^5^
4	1·10^6^
5	4·10^5^
6	7·10^5^
7	6·10^5^
8	5·10^5^
9	1·10^5^

As the problem with defect irregularities on the paper was mainly on PM1 (as informed by the paper mill), we assessed the bacterial communities in the water circulations of all paper machines to compare them and identify differences. The clear filtrates are well filtered and used to prepare the raw material (e.g. pulp fiber) and, as such, may enter the circulation of all four paper machines. The two recycled waters from the wire section (white water) and from the press section (press water) are turbid waters that are, for the majority, recycled continuously for wet end fiber stock preparations.

The samples taken from the recycled waters from the paper machines showed a moderate bacterial contamination, as determined by culturing methods and quantitative PCR of total DNA (Table 2). In addition to the total DNA extraction from the waters, we treated the samples prior to DNA extraction with PMA to assess the fraction of DNA arising from live bacteria (Table 2). The PMA treatment removed the free DNA from dead cells and reduced the DNA values measured in all samples compared to the total DNA fraction.

**Table 2:** Quantification of bacterial counts in water samples

**A**	Total viable count [cfu^.^cm^−3^]			
	PM1	PM2	PM3	PM4
Clear filtrate	>10^3^	>10^3^	>10^3^	>10^4^
White water	>10^4^	>10^5^	>10^4^	>10^4^
Press water	>10^4^	>10^4^	>10^5^	>10^4^

**B**	Total DNA [genome equivalents cm^−3^]			
	PM1	PM2	PM3	PM4
Clear filtrate	2·10^3^	4·10^3^	6·10^4^	5·10^3^
White water	3·10^4^	3·10^4^	1·10^5^	4·10^4^
Press water	3·10^3^	1·10^4^	5·10^3^	5·10^3^

**C**	PMA‐treated DNA (live) [genome equivalents cm^−3^]			
	PM1	PM2	PM3	PM4
Clear filtrate	1·10^3^	3·10^3^	6·10^3^	5·10^3^
White water	1·10^4^	2·10^4^	1·10^4^	3·10^3^
Press water	8·10^2^	1·10^4^	3·10^3^	2·10^3^

(A) Total viable count as colony‐forming units (cfu) per cm^3^. (B) Total bacterial DNA. (C) DNA from live bacteria. For live fraction, the samples were PMA‐treated prior DNA isolation and quantification to remove DNA from dead bacteria. Bacterial DNA was quantified by 16S real‐time PCR relative to a standard consisting of genomic DNA equivalents of *E. coli* K1.

**Figure 3:**
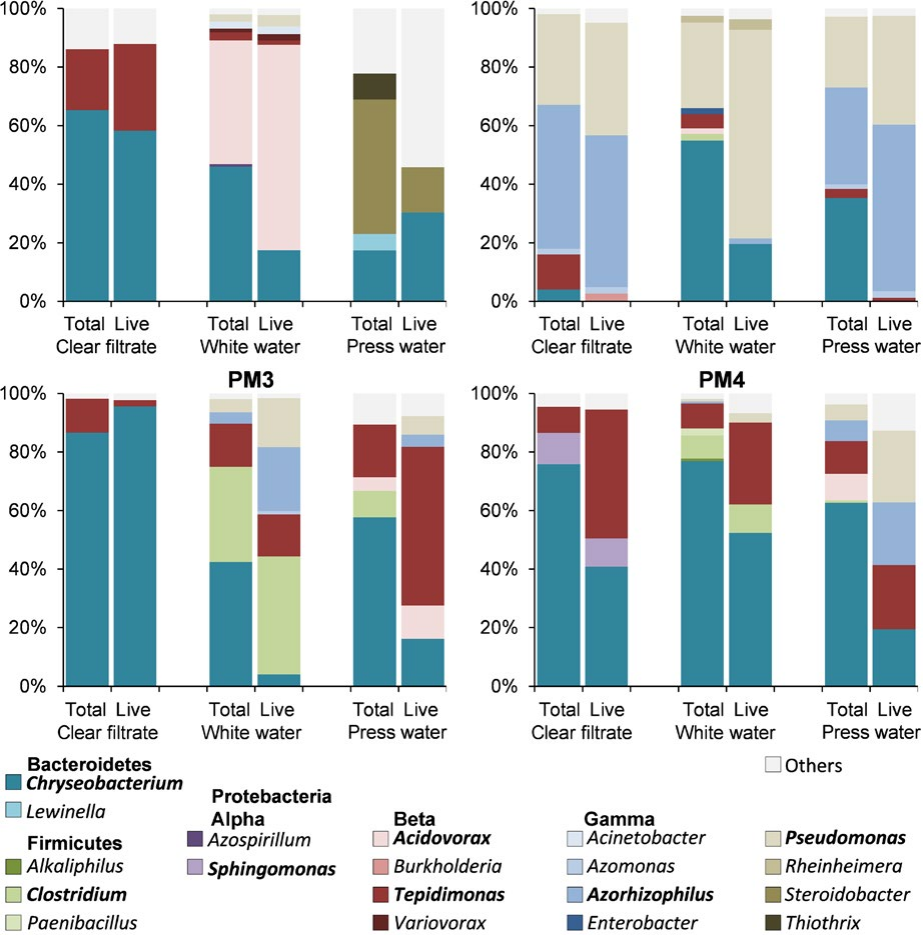
Bacterial population, identified by 16S rRNA metagenomics analysis, in process waters of the four different paper machines (PM1‐PM4) located at the same paper plant. For each sample, the **total** bacterial population and the PMA‐treated fraction, representing the **live** bacterial population, were quantified

The total and PMA‐treated (live) DNA samples were subsequently used to identify and quantify the genera present in the bacterial community (Figure 3). There were only minor differences apparent between the genus diversity determined using total DNA and PMA‐treated DNA. The proportions of the genera varied between the two DNA preparation methods, but the main genera show up in both samples, as shown in Figure 3. The number of abundant genera (i.e. at least 0.5% of all classified genera) correlates between the live and total DNA sample with linear correlation coefficient of *R* ^2^ =* *0.82. Table S2 displays the number of the abundant genera identified in the different samples as well as the calculated Shannon's diversity (Shannon & Weaver, 1946), evenness, and statistical data of the analysis.

Even though all samplings were from the same mill, the bacterial diversities were, nonetheless, unique for each paper machine and sample type. This confirms previous observations showing the unique bacterial population in different paper machines and mills (Granhall et al., 2010). Nevertheless, several similarities between the machines and samples became apparent.

The most distinct bacterial population appears in the samples from PM2, with members of the Gammaproteobacteria predominating in all waters where the genera *Pseudomonas* and *Azorhizophilus* are dominating. PM1, PM3, and PM4 mainly harbor members of the Bacteroidetes and Betaproteobacteria. Abundant genera besides *Chryseobacterum*,* Tepidimonas*, and *Acidovorax* which are discussed below were *Clostridium*,* Pseudomonas*, and *Steroidobacter*. The genus *Pseudomonas* is vast and consists of many environmental bacteria that can be basically found in every habitat (Peix, Ramírez‐Bahena, & Velázquez, 2009). The genus *Clostridium* was mainly found in the white water of PM3. They are anaerobic and endospore forming and were found in diverse environments (Rodloff, 2005). Of the genus *Steroidobacter* found in PM1, only one species could be found was *Steroidobacter denitrificans*. It was isolated from wastewater of a wastewater treatment plant (Fahrbach et al., 2008).

Interestingly, the two genera *Chryseobaterium* and *Tepidimonas*, identified as causative factors for bad paper quality from PM1, could also be identified in all other paper machines. Especially in the water cycle of PM3 and PM4 the two genera represented the majority of all the classified genera. In PM1, these two genera were a minority in the two immediate recycled turbid waters (white water and press water). On the other hand, the clear filtrate, which is heavily reduced in particles, and represents water leaving the PM1 to be reused for all paper machines, showed predominantly the two troubling genera. Different possibilities could account for the seemingly contradicting results. First, PM1 experienced more frequent maintenance periods due to the defective paper sheets. These different frequencies could influence the bacterial population. The nearly complete absence of *Tepidimonas* spp. in the white and press water was, however, very surprising, as the defect problems remained after maintenance. Even more surprising is that although *Tepidimonas* spp. were the most abundant genera in slime deposits on the paper sheets of PM1, they were found to be present in all clear filtrates used for the raw material preparation (e.g., pulping) and abundantly identified in all waters of the smoothly running paper machines PM3 and PM4. One explanation could be that *Tepidimonas*, together with *Chryseobacterium*, grow as compact biofilms and slimes in PM1 exclusively due to an unknown trigger. This would then lead to defect paper due to deposit of the slime. When these slimes dislocate, they remain in the paper web. As such, by far the majority of bacterial cells present in the biofilm (i.e., *Tepidimonas* sp.) would be filtered out by the paper web and not enter the white and press water. Such a trigger for film formation could be the identified species *Acidovorax*, mainly identified in PM1 white water. This genus was shown to be an important colonizer of the head box adapted to the available carbon sources (Kashama, Prince, Simao‐Beaunoir, & Beaulieu, 2009) and abundant in activated sludge communities (Willems & Gillis, 2005). It is known for its aggregating abilities due to generation of an extracellular DNA matrix for attachment (Heijstra, Pichler, Liang, Blaza, & Turner, 2009). As such *Acidovorax* sp. contribute to young biofilms (Liu et al., 2012). It is very well possible that *Chrysobacterium* sp. and *Tepidimonas* spp. require the extracellular matrix produced by *Acidovorax* sp. to generate compact slimes, and, as such, cause the paper defects. The bacteria cells of *Acidovorax* sp., however, are not part of the slime. This is consistent with Kolari et al. (2001) who showed that *Bacillus* sp. uses *Deinococcus geothermalis* as an auxiliary factor to form biofilms in paper machines. Interestingly, some *Bacillus* species then emit heat‐stable metabolites in order to inhibit the growth of *Deinococcus geothermalis*. This could explain that we did not find *Acidovorax* sp. in our samples as it was suppressed by the two later colonizers. Another explanation is that *Acidovorax* sp., as a primary colonizer is present in PM1 due to the more frequent maintenances and that the trigger for the biofilm formation is due to another factor.

Although this study offers an overview of the likely contributory bacterial factors in slime formation, besides not investigating replicate samples, a vital remaining factor also not investigated here is the substrate and environment upon which the slime is formed. Surface morphology, surface chemistry, and physical conditions such as normally stagnant regions in water flows occasionally exposed to shear flow and/or presence of vibration encouraging detachment, as well as oxygenation and moisture levels, exposure to biocide concentration variations, etc., all contribute to the impact of biofilm and slime in sensitive processes such as papermaking.

As a conclusion we can say that as *Tepidimonas* spp. was found in all paper machines, the development of problematic slimes is obviously not only dependent on the mere presence of given bacteria in a system. Auxiliary factors generating the necessary environment, possibly other bacterial species, can be as important. A thorough process analysis for the bacterial communities present helps to shed light on critical factors controlling slime formation. In the present case, targeting *Chrysobacterium* sp. and *Tepidimonas* spp. would bring little success as they are present in all paper machines, and even in the clear filtrate. However, the established bacterial population at the process steps is indicators for the given environmental conditions. Such differences as seen between the paper machines (PM1‐4) are recommended as the points of action to change the environmental conditions for the good (e.g. aeration, stirring adaption). The success of modifications to a favorable microbial community and environment can again be followed by population analysis.

## Conflict of Interest

No conflict of interest declared.
