# Supplementary material for: Characterizing bacterial communities in paper production—troublemakers revealed
Source: Microbiologyopen. 2017 May 14;6(4):e00487. doi: 10.1002/mbo3.487 (PMC5552955; doi:10.1002/mbo3.487)
Supplement: Supplementary file 1 [file MBO3-6-na-s001.pdf]

Table S1. Sample description.

| ID | Paper machine | Sampling site | Constitution |
|---|---|---|---|
| - | PM1 | *Clear filtrate* | liquid |
| - | PM1 | *White water* | liquid |
| - | PM1 | *Press water* | liquid |
| - | PM2 | *Clear filtrate* | liquid |
| - | PM2 | *White water* | liquid |
| - | PM2 | *Press water* | liquid |
| - | PM3 | *Clear filtrate* | liquid |
| - | PM3 | *White water* | liquid |
| - | PM3 | *Press water* | liquid |
| - | PM4 | *Clear filtrate* | liquid |
| - | PM4 | *White water* | liquid |
| - | PM4 | *Press water* | liquid |
| 1 | PM1 | *Paper* | solid |
| 2 | PM1 | *Paper* | solid |
| 3 | PM1 | *Paper* | solid |
| 4 | PM1 | *Paper* | solid |
| 5 | PM1 | *Paper* | solid |
| 6 | PM1 | *Paper* | solid |
| 7 | PM1 | *Paper* | solid |
| 8 | PM1 | *Paper* | solid |
| 9 | PM1 | *Paper* | solid |
