# Supplementary material for: Characterizing bacterial communities in paper production—troublemakers revealed
Source: Microbiologyopen. 2017 May 14;6(4):e00487. doi: 10.1002/mbo3.487 (PMC5552955; doi:10.1002/mbo3.487)
Supplement: Supplementary file 2 [file MBO3-6-na-s002.pdf]

Table S2. Diversity analysis and sequencing Statistics. 16S rRNA metagenomics analysis was performed on an Illumina MiSeq. For diversity analysis, the Shannon's index and Evenness was calculated (Shannon and Weaver, 1946) focusing on genera that represent at least 0.5 % of all classified genera.

		Total Reads	% Reads Passing Quality Filtering	Reads classified at Genus level	Shannon's diversity *	Evenness*	No of genera*
Clear filtrate							
PM1	Total	782185	85.0%	69223	0.89	0.50	6
PM1	Live	664567	86.8%	69580	1.00	0.56	6
PM2	Total	231383	87.6%	121939	1.17	0.73	6
PM2	Live	361476	86.8%	87766	0.99	0.55	5
PM3	Total	249612	88.6%	209250	0.36	0.52	2
PM3	Live	324662	87.0%	188304	0.10	0.14	2
PM4	Total	284820	86.4%	121675	0.76	0.47	5
PM4	Live	121823	85.0%	43345	1.00	0.72	4
White water							
PM1	Total	164484	88.4%	137245	1.12	0.58	7
PM1	Live	289474	88.2%	184742	0.95	0.53	6
PM2	Total	211518	89.6%	178500	1.15	0.59	7
PM2	Live	88417	88.0%	54244	0.86	0.48	6
PM3	Total	205434	88.6%	183884	1.30	0.81	6
PM3	Live	336905	87.2%	210262	1.46	0.81	5
PM4	Total	274173	87.6%	230717	0.85	0.44	7
PM4	Live	838642	86.1%	138963	1.19	0.66	6
Press water							
PM1	Total	535407	86.7%	104297	1.72	0.65	18
PM1	Live	489836	84.7%	19691	2.20	0.76	14
PM2	Total	349053	87.5%	154945	1.33	0.74	6
PM2	Live	204973	87.2%	120709	0.86	0.53	5

PM3	Total	235568	87.1%	44028	1.38	0.60	10
PM3	Live	224485	84.2%	42854	1.32	0.68	7
PM4	Total	384081	87.7%	239897	1.20	0.62	8
PM4	Live	223236	84.3%	31119	1.68	0.81	7
Samples of defect paper							
1		35227	88.6%	31585	0.74	0.53	4
2		105167	88.8%	89928	0.82	0.59	4
3		127347	87.9%	107787	0.63	0.91	2
4		120698	88.2%	104633	0.77	0.43	6
5		270993	88.8%	231076	0.60	0.87	2
6		204785	90.0%	180108	0.73	0.53	4
7		266673	90.2%	236992	0.66	0.60	3
8		176464	89.1%	155906	0.65	0.59	3
9		268877	89.9%	206175	0.50	0.72	2

* of genera representing at least 0.5 % of all classified reads at genus level.
